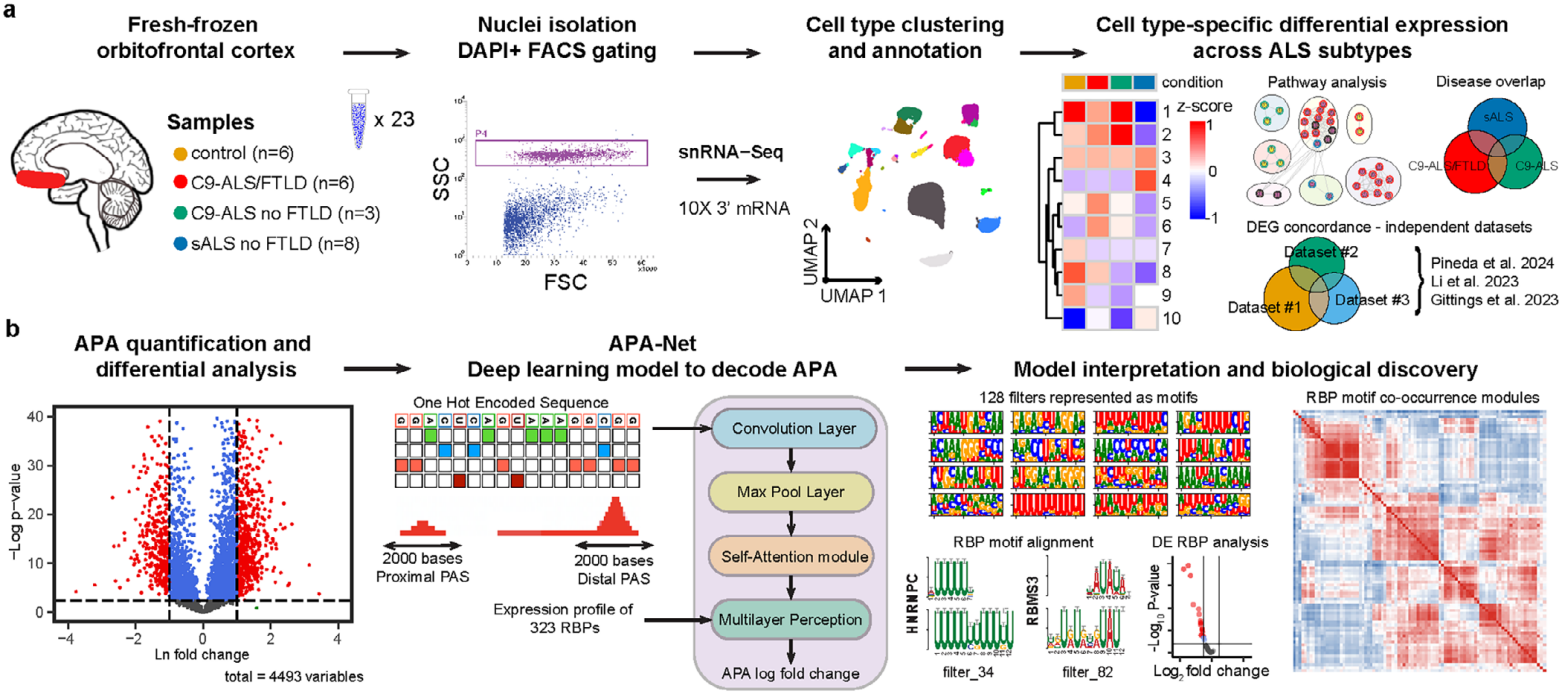# Single‐nucleus transcriptomics and deep learning uncover alternative polyadenylation dysregulation in frontotemporal lobar degeneration and amyotrophic lateral sclerosis

**DOI:** 10.1002/alz70855_103455

**Published:** 2025-12-23

**Authors:** Paul M McKeever

**Affiliations:** ^1^ Tanz Centre for Research in Neurodegenerative Diseases, Toronto, ON, Canada

## Abstract

**Background:**

Frontotemporal lobar degeneration (FTLD) is a leading cause of dementia, often co‐occurring with amyotrophic lateral sclerosis (ALS). Despite shared genetic and pathological features, the cell type‐specific molecular mechanisms underlying FTLD in ALS remain poorly understood.

**Method:**

Excitatory neurons exhibited synaptic dysfunction in ALS with and without FTLD, while inhibitory neurons were more affected in ALS without FTLD. Microglia displayed a disease‐associated state in both groups, with ALS with FTLD showing elevated JAK‐STAT signaling, indicative of a proinflammatory phenotype. We identified widespread shifts toward distal 3’UTR usage in ALS with and without FTLD, implicating APA dysregulation. APA‐Net revealed coordinated RBP interactions, including TDP‐43, HNRNPA1, and MBNL1, as key regulators of APA in ALS with and without FTLD. These findings highlight distinct and overlapping molecular signatures between the two disease subtypes.

**Result:**

We identified cell type‐specific transcriptional dysregulation, with excitatory neurons showing synaptic dysfunction in both C9‐ALS and sALS, while inhibitory neurons were more affected in sALS. Microglia exhibited a disease‐associated state, with C9‐ALS microglia showing elevated JAK‐STAT signaling, indicative of a proinflammatory phenotype. We detected widespread shifts toward distal 3’UTR usage in ALS/FTLD, implicating APA dysregulation. APA‐Net revealed coordinated RBP interactions, including TDP‐43, HNRNPA1, and MBNL1, as key regulators of APA in ALS/FTLD. These findings highlight distinct and overlapping molecular signatures in C9‐ALS and sALS, with implications for FTLD pathogenesis.

**Conclusion:**

This study provides a comprehensive single‐nucleus transcriptomic atlas of the frontal cortex in ALS with and without FTLD, uncovering cell type‐specific dysregulation of APA and RBP interactions. Our findings offer new insights into the molecular mechanisms driving FTLD and provide a resource for future therapeutic development.